# The use of *Euphorbia hirta* L. (Euphorbiaceae) in diarrhea and constipation involves calcium antagonism and cholinergic mechanisms

**DOI:** 10.1186/s12906-019-2793-0

**Published:** 2020-01-16

**Authors:** Muhammad Zeeshan Ali, Malik Hassan Mehmood, Muhammad Saleem, Anwarul-Hassan Gilani

**Affiliations:** 1 Department of Pharmacology, Faculty of Pharmaceutical Sciences, Government College University, Faisalabad, Pakistan; 2 College of Pharmacy, Department of Pharmacology, University of Punjab Old Campus, Lahore, Pakistan; 3 The University of Haripur, Haripur, Khyber Pakhtunkhwa Pakistan

**Keywords:** *Euphorbia hirta*, Antidiarrheal, Ca^++^ antagonist, Laxative, Cholinergic receptor agonist

## Abstract

**Background:**

*Euphorbia hirta* (Linn) family Euphorbiaceae has been used in indigenous system of medicine for the treatment of gastrointestinal disorders. This study was designed to determine the pharmacological basis for the medicinal use of *E. hirta* in diarrhea and constipation.

**Methods:**

The aqueous-methanol extract of whole herb of *E. hirta* (EH.Cr) and its petroleum ether (Pet.EH), chloroform (CHCl_3_.EH), ethyl acetate (Et.Ac.EH) and aqueous (Aq.EH) fractions were tested in the in-vivo experiments using Balb/c mice, while the in-vitro studies were performed on isolated jejunum and ileum preparations of locally bred rabbit and Sprague Dawley rats, respectively, using PowerLab data system.

**Results:**

Qualitative phytochemical analysis showed the presence of alkaloids, saponins, flavonoids, tannins, phenols, cardiac glycosides, while HPLC of EH.Cr showed quercetin in high proportion. In mice, EH.Cr at the dose of 500 and 1000 mg/kg showed 41 and 70% protection from castor oil-induced diarrhea, respectively, similar to the effect of quercetin and loperamide, while at lower doses (50 and 100 mg/kg), it caused an increase in the fecal output. In loperamide-induced constipated mice, EH.Cr also displayed laxative effect with respective values of 28.6 and 35.3% at 50 and 100 mg/kg. In rabbit jejunum, EH.Cr showed atropine-sensitive inhibitory effect in a concentration-dependent manner, while quercetin and nifedipine exhibited atropine-insensitive effects. Fractions of *E. hirta* also produced atropine-sensitive inhibitory effects except Pet.EH and CHCl_3_.EH. On high (80 mM) and low (20 mM) K^+^ − induced contractions, the crude extract and fractions exhibited a concentration-dependent non-specific inhibition of both spasmogens and displaced concentration-response curves of Ca^++^ to the right with suppression of the maximum effect similar to the effect quercetin and nifedipine. Fractions showed wide distribution of spasmolytic and Ca^++^ antagonist like effects. In rat ileum, EH.Cr and its fractions exhibited atropine-sensitive gut stimulant effects except Pet.EH.

**Conclusion:**

The crude extract of *E. hirta* possesses antidiarrheal effect possibly mediated through Ca^++^ antagonist like gut inhibitory constituents, while its laxative effect was mediated primarily through muscarinic receptor agonist like gut stimulant constituents. Thus, these findings provide an evidence to the folkloric use of *E. hirta* in diarrhea and constipation.

## Background

Constipation and diarrhea are commonly prevailing gastrointestinal disorders in our society. The major contributing factors include lack of fibrous diet intake, stress, insufficient fluid intake, excessive use of chemical drugs, smoking and sedentary lifestyle. Constipation is more prevalent in elderly women and men [1]. Diarrhea is one of major cause of morbidity and mortality in developing countries being more common in infants. Around two and half million children die each year worldwide due to diarrhea, 80% of these are reported from developing countries [2]. Medicinal plants may be preferred over standard therapeutic agents to treat these disorders because of the presence of varied phytochemical constituents with synergistic and/or adverse effect neutralizing potential, thus proven to be relatively safe for prolonged use [3].

*Euphorbia hirta* (Linn), family Euphorbiaceae [4] is used in traditional system of medicine for gastrointestinal disorders like diarrhea, dysentery, constipation, intestinal parasites, heartburn, nausea, vomiting, colics and peptic ulcers [5–7]. *E. hirta* and its fresh milk latex are also popular for its medicinal use in bronchial asthma, kidney stones, cold, skin and mucous membranes ailments (wounds, warts, tinea, scabies, thrush, measles, aphthae, and fungal afflictions), microbial infection, conjunctivitis, headache, toothache and rheumatism [4, 8].

Phytochemical studies of *E. hirta* indicated the presence of afzelin, quercitrin [9], myricitrin, rutin, euphorbin-A, euphorbin-B, euphorbin-C, euphorbin-D, 2,4,6-tri-*O*-galloyl-β-d-glucose, euphorbianin, leucocyanidol, camphol, 1,3,4,6-tetra-*O*-galloyl-β-d-glucose, kaempferol, gallic acid, palmitic acid, niacin or nicotinic acid, protocatechuic acid, β-amyrin, 24-methylenecycloartenol, β-sitosterol, heptacosane, shikmic acid, tinyatoxin, choline [8] benzoic acid, caffeic acid and epicatechin 3-gallate acid [10, 11].

*E. hirta* has been reported to possess numerous pharmacological activities such as, antioxidant, anti-inflammatory and anticancer [12], wound healing potential [13], anthelmintic, angiotensin converting enzyme inhibitory, antidipsogenic, antiarthritis and galactogenic [8], antibacterial [14], antianaphylactic [15], antidiabetic [16], anxiolytic [17] and hepatoprotective [13, 18]. In support of the dual medicinal use of *E. hirta* in opposing gut disorders like diarrhea and constipation, previous reports have shown unclear findings like, Tona et al. [19] revealed non-specific antispasmodic action of *E. hirta* in the in vitro. While, Hore et al. [20] demonstrated only gut inhibitory effects of *E. hirta* in naïve rats and castor oil-administered mice. Galvez et al. [21] reported its gastrointestinal transit delaying potential in castor oil-administered animals. Kumgang et al. [22] showed its gut stimulant effects in the in vitro and antidiarrheal activity in the in vivo. To explain the folk use of *E. hirta* in diarrhea and constipation and to address the paucity in existing literature whether it possesses both laxative and antidiarrheal effects, the primary objective of this study was to determine the pharmacological basis to the medicinal use of *E. hirta* in diarrhea and constipation. While the secondary objective was to estimate the distribution and comparative efficacy of gut stimulant and relaxant constituents in polarity-driven fractions of *E. hirta*. The in-vivo and *in –vitro* assays were conducted on mice and the isolated tissues of rabbits and rats, respectively. Based on their anatomical, physiological and genetic commonalities with human biology, murine models are extensively used in research worldwide to establish the efficacy of test material(s) in diarrhea and constipation [23–26].

## Methods

### Preparation of plant extract and fractions

Whole plant was collected in November, 2017 from the surroundings of Faisalabad, Punjab, Pakistan. The plant was identified by the expert taxonomist Dr. Mansoor Hameed, Associate Professor, Department of Botany, University of Agriculture, Faisalabad. The specimen (voucher no. 415–1-2019) was kept at herbarium of University of Agriculture, Faisalabad. Plant material (6 kg) was shade dried and coarsely powdered in electrically driven cutter. Powdered material was macerated in 5 L of aqueous- methanol (30:70) thrice with occasional forceful shaking. The supernatant(s) were combined and evaporated under reduce pressure in rotary evaporator (Stuart RE 300, Cole-Parmer scientific experts, Staffordshire, ST15 OSA, UK) at 40 °C temperature. The yield of the extract was 7.3% w/w. For fractionation, EH.Cr was dissolved in 150 mL of distilled water and fractioned with petroleum ether, chloroform and ethyl acetate into a separating funnel. Organic fractions were evaporated while the aqueous fraction was lyophilized [27].

### Phytochemical analysis

The crude extract of *E. hirta* was tested for alkaloids, flavonoids, saponins, tannins, phenols and glycosides by following the standard methods [28].

### HPLC fingerprinting of *E. hirta* extract and its fractions

High performance liquid chromatography of EH.Cr and its fractions along with four standard compounds (kaempferol, quercetin, benzoic acid and rutin) sourced from Sigma Chemicals Co., St. Louis, MO, USA, was performed using a Shimadzu (Japan) Prominence 10-AT equipped with a pump (LC-10AT), PDA detector (SPD-10AV) and column [Shim-Pack CLC-ODS (C-18), 25 cm × 4.6 mm, 5 μm]. The samples were prepared in methanol and were passed through a Millipore filter (2.4 μ) prior to inject using a micro syringe. The mobile phase containing acetonitrile, methanol and acetic acid was delivered at a flow-rate of 1 mL/min. The column temperature was maintained at 25 °C. UV –visible detector measured at wavelength of 280 nm.

### Experimental animals

Locally bred rabbits (1–1.5 kg), Bulb/c mice (20-25 g) and Sprague–Dawley rats (200–250 g) of either sex were used in this study. The animals were maintained in the animal house at 23 ± 5 °C and 55 ± 5% relative humidity with 12-h light/dark cycle. The animals were housed in plastic cages with sawdust bedding material (replaced thrice in a week). The rabbits were provided tap water and fresh green fodder ad libitum, while thee mice and rats were given tap water and the standard diet containing g/Kg, flour 380; choker 380; NaCl 5.8; molasses 12; nutrivet L 2.5; vegetable oil 38; potassium meta bisulphate 1.2; powder milk 150 and fish oil 170. Animals were acclimatized for 5 to 7 days before starting experiment and kept on overnight fasting with free water access before the experiment. All the experiments were conducted in the morning h (in light phase only), while animals were monitored carefully for any unusual behavioral changes [25]. The study protocols were approved by the Institutional Review Board, Government College University, Faisalabad, Pakistan with reference no. GCUF/ERC/1983 dated: 27.06.2018. Experiments were carried out by following the guidelines of Institute of laboratory Animal Resources Commission on Life Sciences, National Research Council (1996).

### In-vivo studies

#### Acute toxicity testing

A total of forty healthy adult Bulb/c mice (20–25 g) of either sex were randomly divided by physical randomization in four groups (*n* = 10/group). The first group received saline (10 mL/kg) and the second, third and fourth group were administered 3, 5 and 10 g/kg of *E. hirta* extract, respectively. Thereafter, mice were kept in cages with free access to water and *ad labitum*. The animals were observed at regular intervals for pilo erection, changes in exploratory behavior, locomotor activity, feeding behavior and blindness for 6 h. However the lethality was monitored up to 24 h.

#### Castor oil-induced diarrhea

Healthy adult Bulb/c mice (8–10 weeks old) of either sex weighing, 20–25 g, *n* = 42 were fasted for 12 h with free access to water before experiment. The healthy animals were housed in individual cages and were randomly divided into seven groups (*n* = 6/group) by physical randomization. Physical randomization was carried out as follows; all animals were marked by tail numbering method. All numbers were preserved on slips of paper and folded individually in a box. The box was shaken thoroughly. All slips were withdrawn simultaneously as per number of animals in each group. Each group of animal was labeled alphabetically. One group as negative control received normal saline (10 ml/kg). Second group received 10 mg/kg of loperamide (Highnoon Laboratories Pvt. Ltd., Lahore, Pakistan), orally as positive control. The third and fourth groups were administered 500 and 1000 mg/kg of *E. hirta* extract orally, while fifth to seventh groups received 50, 100 and 150 mg/kg of quercetin through oral route, respectively. After 1 h of treatment, all the groups received castor oil (Care Pharmacy, Faisalabad, Pakistan) 10 ml/kg, orally through gastric feeding needle. After 4 h, the cages were inspected to record total number of feces (number of dry feces and wet feces) [28] and the % protection from diarrhea was determined as = 100 – [total number of wet feces/number of total feces × 100].

#### Determination of laxative activity

Healthy adult Bulb/c mice of 8–10 weeks of age (weighing; 20–25 g, *n* = 60) of both sex were fasted for 12 h with free access to water before the experiment. The healthy animals were placed individually in clean cages lined with filter paper and were randomly divided by physical randomization into 10 groups (*n* = 6/group). Each group of animal was labeled alphabetically. The first group (negative control) was administered saline orally (10 ml/kg) and the second group was administered carbachol [CCh, 1 mg/kg intra-peritoneal (i.p)] as positive control. CCh was purchased from Sigma Chemicals Co., St. Louis, MO, USA. The third to fifth groups received 50, 100 and 300 mg/kg of the crude extract of *E. hirta*. Remaining groups were pretreated with atropine (10 mg/kg, i.p.) before re-evaluation of the laxative effects of CCh and different doses of the pant extract. After 6 h, total fecal production (total number of wet feces plus total number of dry feces) was measured and the % age of the wet feces [% laxation = total number of wet feces/number of total feces × 100] was considered as laxative effect [3].

#### Loperamide-induced constipation

Healthy adult Bulb/c mice weighing 20–25 g, *n* = 24 (8–10 weeks old) were divided into four groups, *n* = 6/group. All the animals were kept in individual cages. The animals in first group served as negative control and received saline (10 mL/kg). The second to fourth groups received loperamide (5 mg/kg) orally 1 h prior to any treatment. The second group served as disease control and received no treatment, while animals of treatment groups (third and fourth) were administered the crude extract of *E. hirta* (50 and 100 mg/kg, orally). After 6 h, total feces production (total number of wet feces plus total number of dry feces) was measured and the percentage of the wet feces [% laxation = total number of wet feces/number of total feces × 100] was considered as laxative effect [29].

### In-vitro studies

#### Preparation of isolated rabbit jejunum segments

The healthy adult rabbits (6–7 months old) were selected randomly for the study and anaesthetized using intraperitoneal injection of thiopental sodium at dose range of 70–100 mg/kg. Sodium thiopental was purchased from Care Pharmacy, Faisalabad, Pakistan. Once rabbit was anaesthetized and confirmed by absence of touch and corneal reflexes, thoracotomy was performed followed by cardiac puncture to euthanize the animal. The abdomen was cut open using sharp edged blade and the jejunum was isolated and immersed in Tyrode’s solution in petri dish aerated with carbogen (95% O_2_ and 5% CO_2_). The mesenteries were carefully removed. Individual segments of jejunum (2–3 cm) were hanged in 10 mL tissue organ baths containing Tyrode’s solution maintained at 37 °C and aerated by carbogen. Tyrode’s solution contained KCI; 2.68, NaCI; 139.9, MgCI_2_; 1.05, NaHCO_3_; 11.90, NaH_2_PO_4_; 0.42, CaCl_2_; 1.8 and Glucose; 5.55 in mM with a pH 7.4. Administered dose of thiopental sodium did not affect normal activity pattern of isolated tissue in tissue organ bath. The response of the crude extract on intestinal preparations was recorded using the isometric force transducer coupled with the PowerLab data system (PowerLab version ML4/25, ADInstruments, Australia). A preload of 0.7–1.0 g was applied to individual tissue. Tissues were allowed to equilibrate for at least 30 min before the addition of any drug and then stabilized with repeated administration of acetylcholine (Ach, 0.3 μM, purchased from Sigma Chemicals Co., St. Louis, MO, USA) at 3–5 min interval, until similar responses were achieved. Rabbit jejunum exhibits spontaneous rhythmic contractions thus allows the assessment of spasmolytic activity directly without using an agonist. Inhibitory effect of the test substance was measured as percent change in spontaneous contraction of jejunum [1].

#### Determination of spasmolytic activity

To explore the possible mechanism of spasmolytic effect, the test material was studied on high K^+^ (80 mM) and low K^+^ (20 mM)-induced contractions. To determine the inhibitory effect mediated through Ca^++^ channel blocking like mechanism, the test material was administered to the depolarized by high K^+^ (80 mM). High concentration of calcium (K^+^ ≥ 80 mM) is established to cause contractions in smooth muscles through opening of voltage-dependent Ca^++^ channels which allow the influx of extra-cellular Ca^++^ resulting in contractile response [30, 31]. Any test substance which inhibits high K^+^-mediated contraction is considered a blocker of Ca^++^ influx through L-Type Ca^++^ channels [27]. Once spasmogen-sustained contraction was achieved almost in 5–10 min, the test substance was then added in cumulative order to assess the inhibitory effect. To confirm Ca^++^ channel blockade like effect of the test material, jejunum tissues were stabilized in calcium free Tyrode’s solution, containing EDTA (0.1 mM), to remove the extracellular calcium from the tissue. Further, Ca^++^ free Tyrode’s solution was replaced with K^+^ rich and Ca^++^ free Tyrode’s solution [KCl; 50, NaCl; 91.03, MgCl_2_.6H_2_O; 0.50, NaH_2_PO_4_.2H_2_O; 0.32, NaHCO_3_; 11.9, Glucose; 5.05 and EDTA; 0.1 in mM] to remove the intracellular Ca^++^ [32]. After incubation period of 30 min, a control concentration-response curve (CRC) of Ca^++^ was constructed. When two control CRCs of Ca^++^ were found superimposed (usually after two or three circles), the jejunum was pretreated with increasing concentrations of the test substance for 1 h and the CRCs of Ca^++^ were reconstructed to attest the presence of Ca^++^ antagonist like effect [33].

#### Preparation of rat ileum

The healthy adult Sprague–Dawley rats (8–12 weeks old) were randomly selected and starved for 12–16 h. Rats were anesthetized using isoflurane (2–5% v/w) through inhalation in a closed chamber until fully anesthetized. Isoflurane was purchased from Care Pharmacy, Faisalabad, Pakistan. Once animals were fully anaesthetized and confirmed by absence of touch and corneal reflexes, thoracotomy was performed followed by cardiac puncture to euthanize the animals. The abdomen was cut open using sharp edged blade and the ileum was isolated. Individual ileum segments of 2–3 cm were suspended in 10 mL tissue organ baths containing Tyrode’s solution, maintained at 37 °C and aerated by carbogen. A preload of 0.7–1.0 g was applied to individual tissue. Each tissue was allowed to equilibrate for at least 30 min before the addition of any drug and then stabilized with repeated administration of acetylcholine (Ach, 0.3 μM) at every 3–5 min interval, until similar responses were achieved. Exposed dose of isoflurane did not alter repose of Ach on isolated tissue in tissue organ bath. Once stabilized, the responses of crude extract were recorded using isometric force transducers coupled with PowerLab system [34].

#### Determination of spasmogenic activity

To characterize the possible underlying mechanism of the determined spasmogenic activity, contractile effect of the test material was studied in the absence and presence of atropine (0.1 μM, a cholinergic antagonist) or pyrilamine (1 μM, a histaminergic receptor antagonist) or methysergide (1 μM, a serotonergic antagonist). All antagonists were purchased from Sigma Chemicals Co., St. Louis, MO, USA. The complete blockade of stimulatory effect of the test material in the presence of aforementioned antagonist(s) identified the nature of the constituents in test material mediating gut stimulant effect [25].

### Statistical analysis

Values were expressed as mean ± Standard Error of Mean (S.E.M) and “n” indicated number of experiments /animals used. EC_50_ values represent 50% of the maximal effective concentration with 95% confidence intervals (CIs). Student *t*-test, One-way analysis of variance (ANOVA) followed by Dunnet’s test and One-way ANOVA followed by Bonferroni test were applied for differentiation of data for laxative and antidiarrheal activities. *P* < 0.05 was considered significantly different. Concentration-response curves (CRCs) were analyzed by non–linear regression. Two-way ANOVA followed by Bonferroni’s post-test correction was used for comparison of Ca^++^ CRCs constructed in the absence and presence of test material(s). Gut stimulant effects of test material were compared with baseline using One-way ANOVA followed by Dunnet’s test. Graphs were prepared using Graph Pad program (San Diego, Calf. USA).

## Results

### Phytochemical analysis

Preliminary phytochemical studies of the crude extract of *E. hirta* showed saponins, alkaloids, flavonoids, tannins, phenols and cardiac glycosides as plant constituents.

### HPLC fingerprinting of *E. hirta* extract and its fractions

**Fig. 1:**
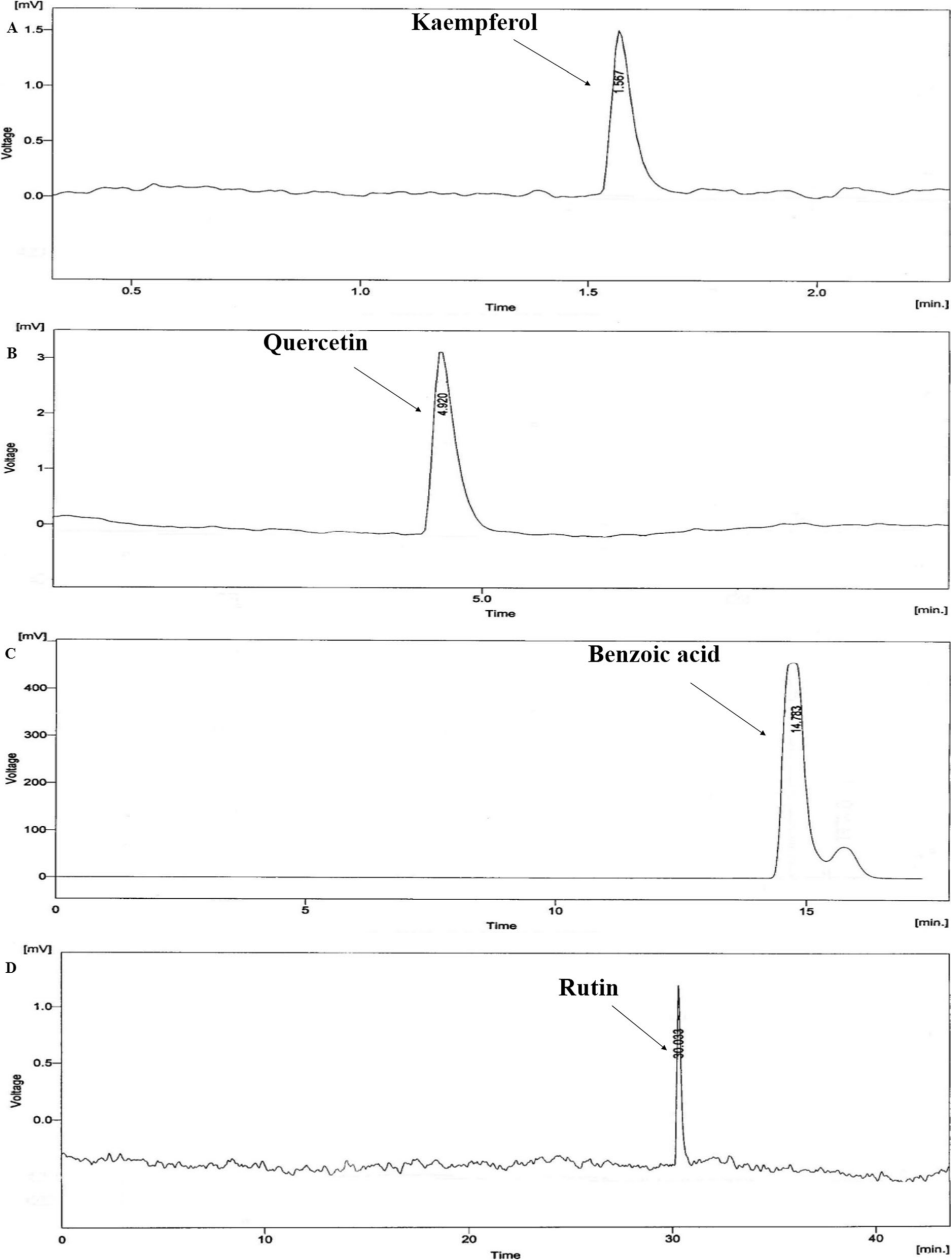
The HPLC chromatogram of (**a**) kaempferol, (**b**) quercetin, (**c**) benzoic acid and (**d**) rutin which are the standard bioactive compounds identified in *E. hirta*. The labelled peaks of figure indicate retention time (in min) of these compounds

**Fig. 2:**
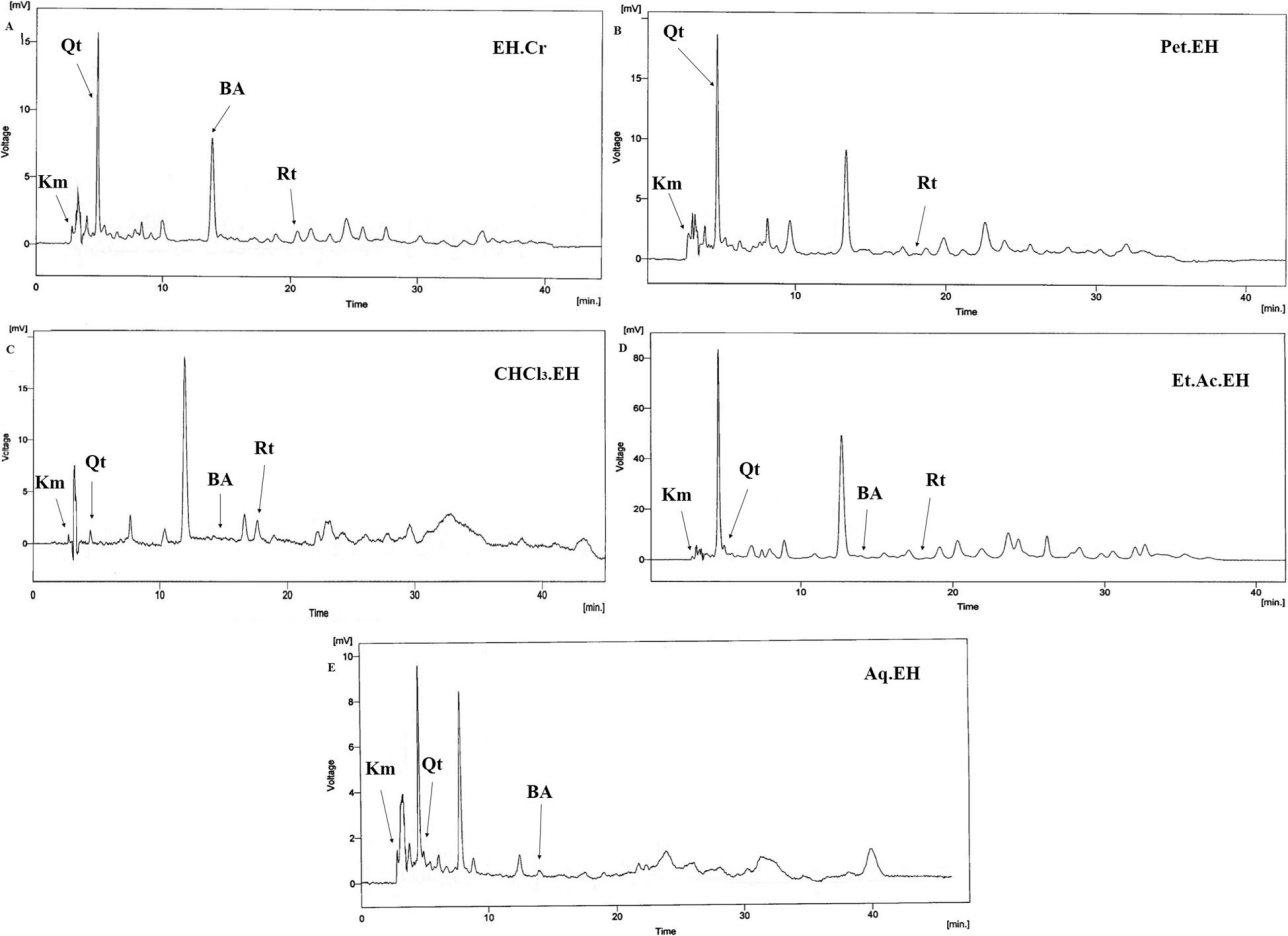
The HPLC chromatogram of (**a**) the crude extract of *E. hirta* and its (**b**) petroleum ether, (**c**) chloroform, (**d**) ethyl acetate and (**e**) aqueous fractions. The peaks of these chromatogram show the presence of kaempferol (Km), quercetin (Qt), benzoic acid (BA) and rutin (Rt) along with other multiple unidentified compounds in the crude extract and its fractions

The HPLC chromatograms of standard compounds; kaempferol (Fig. 1a), quercetin (Fig. 1b), benzoic acid (Fig. 1c) and rutin (Fig. 1d), the crude extract (Fig. 2a), its petroleum ether (Fig. 2b), chloroform (Fig. 2c), ethyl acetate (Fig. 2d), and aqueous (Fig. 2e) fractions were obtained. This chromatogram represented the presence of all four bioactive compounds in the crude extract of *E. hirta,* its chloroform and ethyl acetate fractions. Rutin was found absent in the aqueous fraction, while benzoic acid was absent in the petroleum ether fraction. Quercetin was found in highest concentration in the crude extract of *E. hirta* and its petroleum ether fraction. Numerous unidentified components were also noted in HPLC chromatograms of EH.Cr and its different fractions (Fig. 2).

### In-vivo activity

#### Acute toxicity test

The crude extract of *E. hirta* at the dose of 3, 5 and 10 g/kg was administered to three groups of mice. The animals were monitored for any sign of acute toxicity or lethality for 24 h after the administration of plant extract. The *E. hirta* extract was found safe and devoid of any sign of behavior change like pilo erection, changes in exploratory behavior, locomotor activity, feeding behavior, blindness and the lethality in all the tested groups.

#### Castor oil-stimulated diarrhea

The crude extract of *E. hirta* and quercetin produced dose-dependent antidiarrheal effects in mice. Administration of *E. hirta* at the dose of 500 and 1000 mg/kg to mice produced 41 and 70% protection from diarrhea, respectively, while quercetin at the dose of 50, 100 and 150 mg/kg showed 46, 56 and 65% protection, respectively. The positive control of loperamide treated animals showed 100% protection at the dose of 10 mg/kg, while saline treated animals showed only 18% protection as seen in Table 1. During assessment of antidiarrheal activity, no adverse effect was observed with loperamide, castor oil, quercetin or at administered doses of *E. hirta* extract.

**Table 1:** Antidiarrheal effect of *E. hirta* extract against castor oil-induced diarrhea in mice

Group no.	Treatment	Dose (mg/kg)	Mean feces/group (numbers)	Mean wet feces/group (numbers)	% age protection from diarrhea
1	Saline + castor oil (10 mL/kg)	–	8.5 ± 1.78	6.82 ± 0.47	18%
2	Loperamide + castor oil	10	2.83 ± 0.60 **	0 ± 0.00 **	100%
3	EH Cr. (p.o) + castor oil	500	4.83 ± 0.40 *	2.85 ± 1.24*	41%
4	EH Cr. (p.o) + castor oil	1000	3.84 ± 0.54 **	1.16 ± 1.16**	70%
5	Quercetin + castor oil (p.o)	50	8.16 ± 053	4.16 ± 1.07*	46%
6	Quercetin + castor oil (p.o)	100	7.66 ± 0.61	3.33 ± 0.91*	56%
7	Quercetin + castor oil (p.o)	150	7.33 ± 0.80	2.50 ± 1.41*	65%

*Abbreviations*: *EH.Cr* the crude extract of *E. hirta*, *p.o*. per oral

Values shown are mean ± SEM n = six animals/group. *p<0.05 and **p<0.01 show a comparison of group 2–7 versus group 1 (One way ANOVA followed by Dunnet’s test)

#### Laxative activity

Administration of *E. hirta* to mice resulted in 40.9 and 58.8% production of wet feces at the doses of 50 and 100 mg/kg, respectively, compared to saline group which produced only 7.40% laxation. At higher tested dose of 300 mg/kg, a decline in the production of wet and total feces was observed, showing the co-existence of laxative (at lower doses) and antidiarrheal (at higher doses) effects. Carbachol (1 mg/kg), the positive control produced 54.3% wet feces. When the crude extract of *E. hirta* was restudied in mice pretreated with atropine (10 mg/kg), the productions of total feces was significantly declined as seen in Table 2. During the study protocol of laxative activity, no adverse effect was observed with carbachol, atropine or administered doses of *E. hirta*.

**Table 2:** Laxative effect of *E. hirta* extract in the absence and presence of atropine in mice

Group no.	Treatment	Dose (mg/Kg)	Mean pellets/group (numbers)	Mean wet pellets/group (numbers)	% Laxation
1	Saline (10 mL/kg)	–	4.5 ± 0.42	0.333 ± 0.21	7.40
2	Carbachol	1	11.6 ± 0.67 **	6.33 ± 1.22 **	54.3
3	EH.Cr (p.o)	50	7.33 ± 0.49 *	3 ± 0.36 **	40.9
4	EH.Cr (p.o)	100	8.5 ± 0.42 **	5.10 ± 0.57 **	58.8
5	EH.Cr (p.o)	300	3.53 ± 0.42	1.66 ± 0.30	28.5
6	Saline (10 mL/kg) + atropine	10	3.11 ± 0.36	0.00 ± 0.00	0
7	Carbachol + atropine (i.p)	1 + 10	6 ± 1.30 ^@@^	1.66 ± 0.33 ^@@^	27.7
8	EH.Cr (p.o) + atropine (i.p)	50 + 10	3.5 ± 0.56 ^@@^	0.83 ± 0.30 ^@^	23.8
9	EH.Cr (p.o) + atropine (i.p)	100 + 10	4.83 ± 1.07^@@^	1.33 ± 0.49 ^@@^	27.5
10	EH.Cr (p.o) + atropine (i.p)	300 + 10	1.33 ± 0.21^ns^	0.33 ± 0.22 ^ns^	25

*Abbreviations*: *EH.Cr* the crude extract of *E. hirta*, *p.o*. per oral, *i.p* intraperitoneal

Values are presented as mean ± SEM, *n* = 6 animals/group. */^@^ *p* < 0.05, **/^@@^ *p* < 0.01 and ns indicates non-significant, * shows comparison of group 2–5 vs group 1, (One-way ANOVA followed by Dunnett’s test), ^@^ shows a comparison of group 7 vs group 2, group 8 vs 3, group 9 vs 4 and group 10 vs 5 (One-way ANOVA followed by Bonferroni test)

#### Loperamide-induced constipation

In loperamide-induced constipation model, *E. hirta* at the dose of 50 and 100 mg /kg produced 28.6 and 35.3% of wet feces in mice, respectively, as seen in Table 3.

**Table 3:** Laxative effect of *E. hirta* extract in loperamide–induced constipated mice

Group no.	Treatment	Dose (mg/kg)	Mean defecation/group	Mean amount of wet feces/group	% Wet feces
1.	Saline (10 mL/kg)	10	8.21 ± 0.45	0.79 ± 0.12	9.63
2.	Loperamide + saline	5 + 10	2.31 ± 0.14^***^	0.20 ± 0.09^*^	8.6
3.	Loperamide + EH.Cr (p.o)	5 + 50	4.16 ± 0.13 ^@@/++^	1.19 ± 0.38 ^ns/ns^	28.6
4.	Loperamide + EH.Cr (p.o)	5 + 100	5.1 ± 0.35 ^@@/++^	1.80 ± 0.31 ^@@/ +^	35.3

Values are presented as mean ± SEM, *n* = 6 animals/group. *p < 0.05 and ***p < 0.001 show a comparison of group no. 2 vs group no. 1 (Students *t*-test). ^@^ *p* < 0.05 and ^@@^ *p* < 0.01 show a comparison of group no. 3 and 4 vs group no. 2. ^+^ *p* < 0.05 and ^++^ *p* < 0.01 show a comparison of group no. 3 and 4 vs group no. 1, ns represents non-significant (One-way ANOVA followed by Dunnett’s test)

### In-vitro activity

#### Effect on rabbit jejunum

**Fig. 3:**
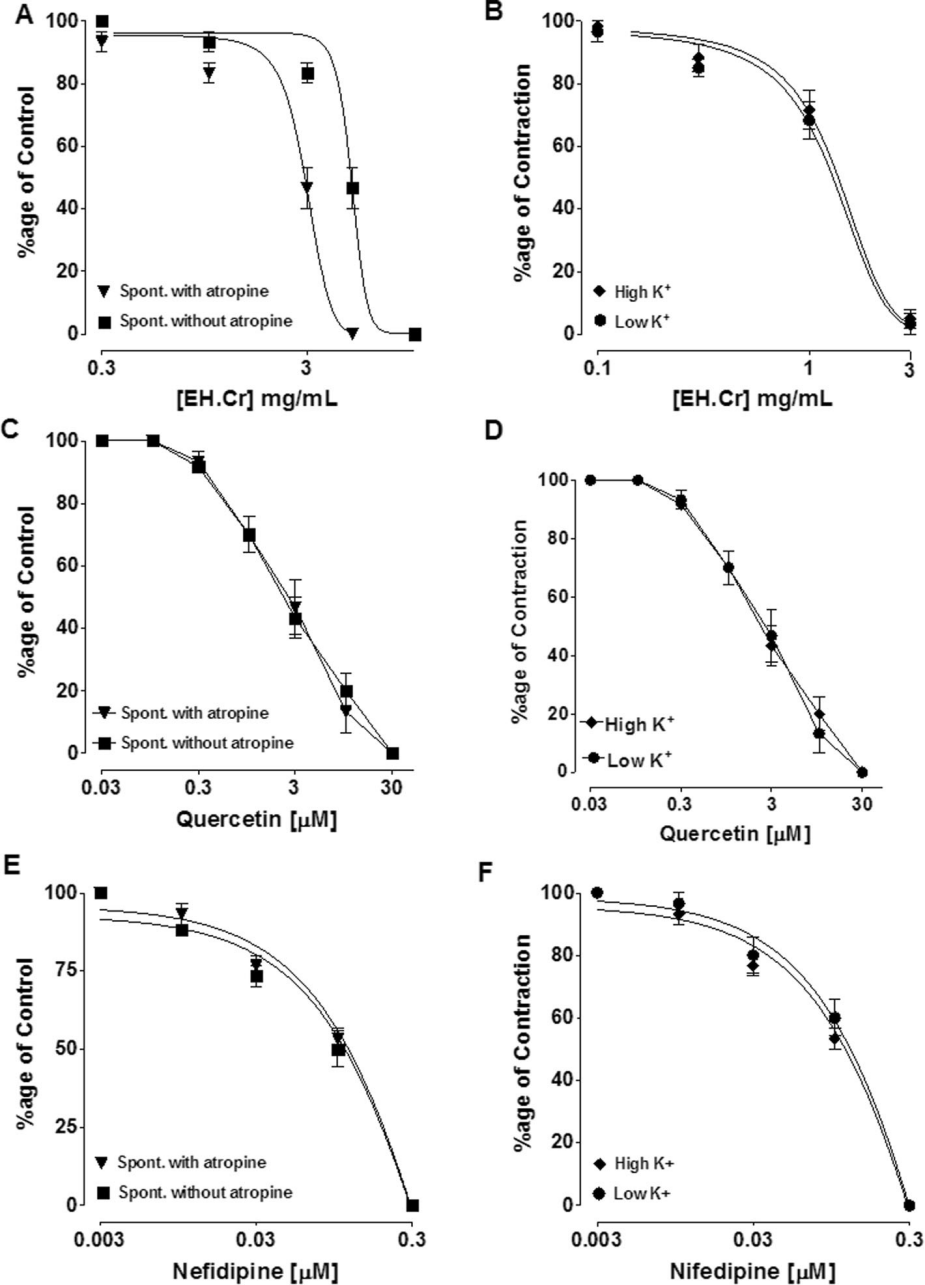
The concentration-dependent inhibitory effect of the crude extract of *E. hirta* (EH.Cr) (**a-b**), quercetin (**c-d**) and nifedipine (**e-f**) against spontaneous (in absence and presence of atropine, (0.1 μM), low K^+^ (20 mM) and high K^+^ (80 mM)-induced contractions on rabbit jejunum. The data shown as mean ± S.E.M, *n* = 4–6 individual experiments using tissues of 4 to 5 animals

**Fig. 4:**
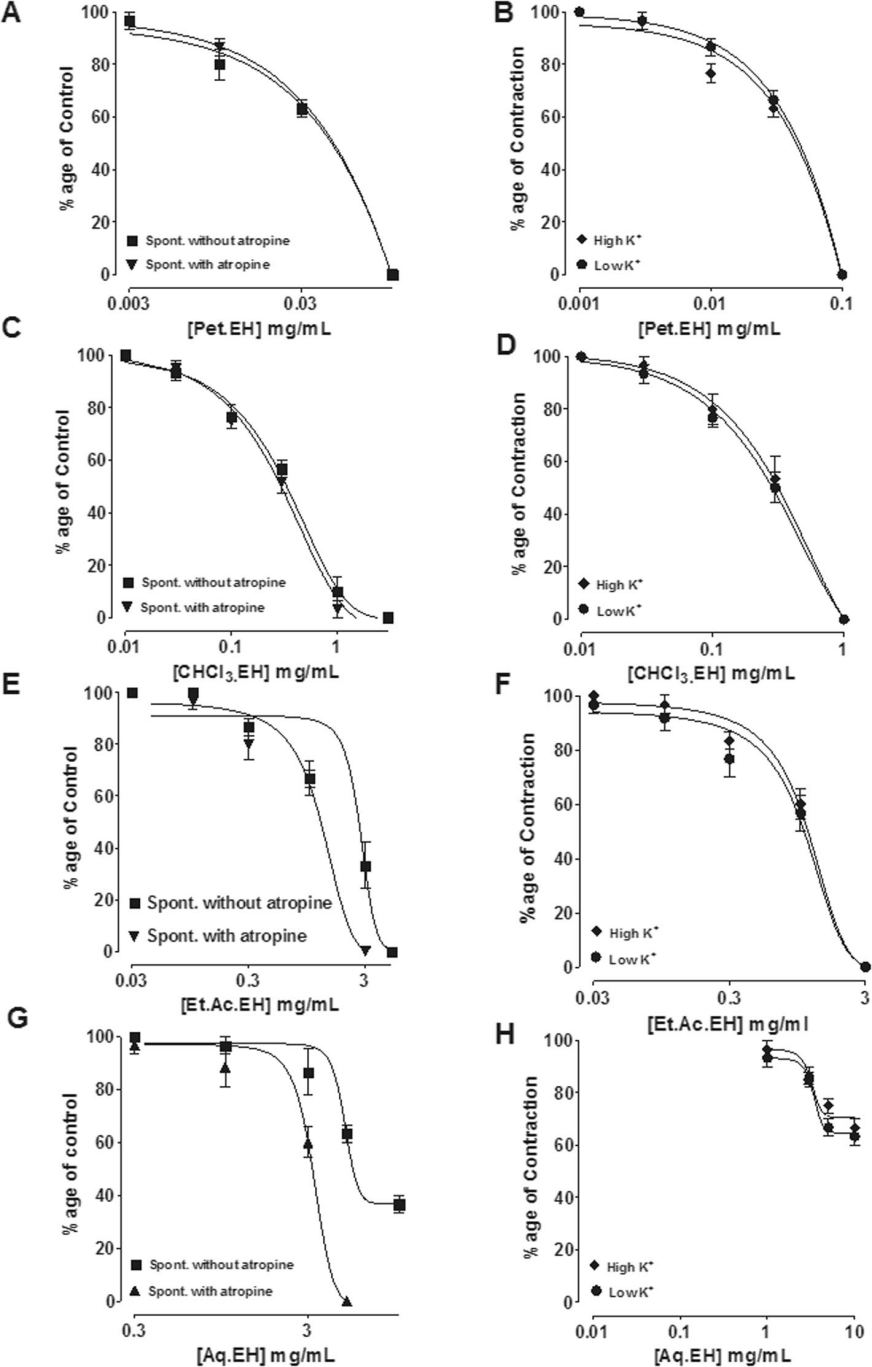
The concentration-dependent inhibitory effect of the petroleum ether (Pet.EH) (**a**-**b**), chloroform (CHCl_3_.EH) (**c**-**d**), ethyl acetate (Et.Ac.EH) (**e**-**f**) and aqueous (Aq.EH) (**g**-**h**) fraction of *E. hirta* against spontaneous [(in absence and presence of atropine, (0.1 μM)], low K^+^ (20 mM) and high K^+^ (80 mM)-induced contractions on rabbit jejunum. The data shown as mean ± S.E.M, *n* = 4–6 individual experiments using tissues of 4 to 5 different animals

The crude extract of *E. hirta* completely relaxed spontaneously contracting rabbit jejunum with EC_50_ value of 5.09 mg/mL (2.72–9.51, 95% CI, *n* = 4–6) in a concentration-dependent manner, (Fig. 3a). The inhibitory effect was potentiated [3.35 mg/mL (1.86–6.03, *n* = 4–6)] in the presence of atropine (0.1 μM) as shown in Fig. 4a. The plant extract inhibited high K^+^ (80 mM) and low K^+^ (20 mM)-induced contractions with any specificity similar to the effect of quercetin and nifedipine in rabbit jejunum (Fig. 3 and Table 4).

**Table 4:** Comparative inhibitory effects of *E. hirta* extract and its fractions in isolated rabbit jejunum

Sr. no.	Test material		EC_50_ (mg/mL) value (95% CI; *n* = 4–6) against spontaneous movement	EC_50_ (mg/mL) value (95% CI; *n* = 4–6) against high K^+^ (80 mM)-induced contractions	EC_50_ (mg/mL) value (95% CI; *n* = 4–6) against low K^+^ (20 mM)-induced contractions
1.	EH.Cr	without atropine	5.09 (2.72–9.51)	1.90 (1.09–3.26)	1.89 (1.07–3.28)
		with atropine	3.36 (1.86–6.03)		
2.	Pet.EH	without atropine	0.28 (0.032–2.43)	0.19 (0.05–0.62)	0.19 (0.06–0.60)
		with atropine	0.29 (0.07–1.08)		
3.	CHCl_3_.EH	without atropine	0.40 (0.26–0.61)	0.83 (0.35–1.95)	0.83 (0.32–1.90)
		with atropine	0.32 (0.21–0.48)		
4.	Et.Ac.EH	without atropine	2.53 (1.59–4.04)	1.38 (0.86–2.19)	1.38 (0.88–2.2)
		with atropine	1.55 (0.88–2.71)		
5.	Aq.EH	without atropine	N/A	N/A	N/A
		with atropine	6.35 (2.78–14.48)		
6.	Quercetin	without atropine	2.57 (1.63–4.03)	2.57 (1.63–4.03)	2.50 (1.55–4.1)
		with atropine	2.57 (1.63–4.03)		
7.	Nifedipine	without atropine	0.13 (0.11–0.44)	0.13 (0.11–0.44)	0.13 (0.11–0.44)
		with atropine	0.13 (0.11–0.44)		

Values showed geometric means with 95% confidence intervals (CI) in parenthesis. “n” shows number of experiments

*Abbreviations*: *EH.Cr* the crude extract of *E. hirta*, *Pet.EH* petroleum ether fraction, *CHCl*_*3*_*.EH* chloroform fraction, *Et.Ac.EH* ethyl acetate fraction, *Aq.EH* aqueous fraction

The ethyl-acetate (Et.Ac.EH) and aqueous (Aq.EH) fractions inhibited the spontaneous contractions, while the observed inhibitory effects were potentiated when studied in the presence of atropine. However, the inhibitory effect of the chloroform (CHCl3.EH) and pet-ether (Pet.EH) fractions remained unchanged when reproduced in the presence of atropine as seen in Fig. 4. Similar to the parent extract, fractions of *E. hirta* inhibited the high and low K^+^-induced contractions without any specificity against high or low K^+^-challenged contractions except the aqueous fraction which showed mild inhibitory effect (Fig. 4). The comparative inhibitory effects of the crude extract and its fractions were represented as EC_50_ values (Table 4).

**Fig. 5:**
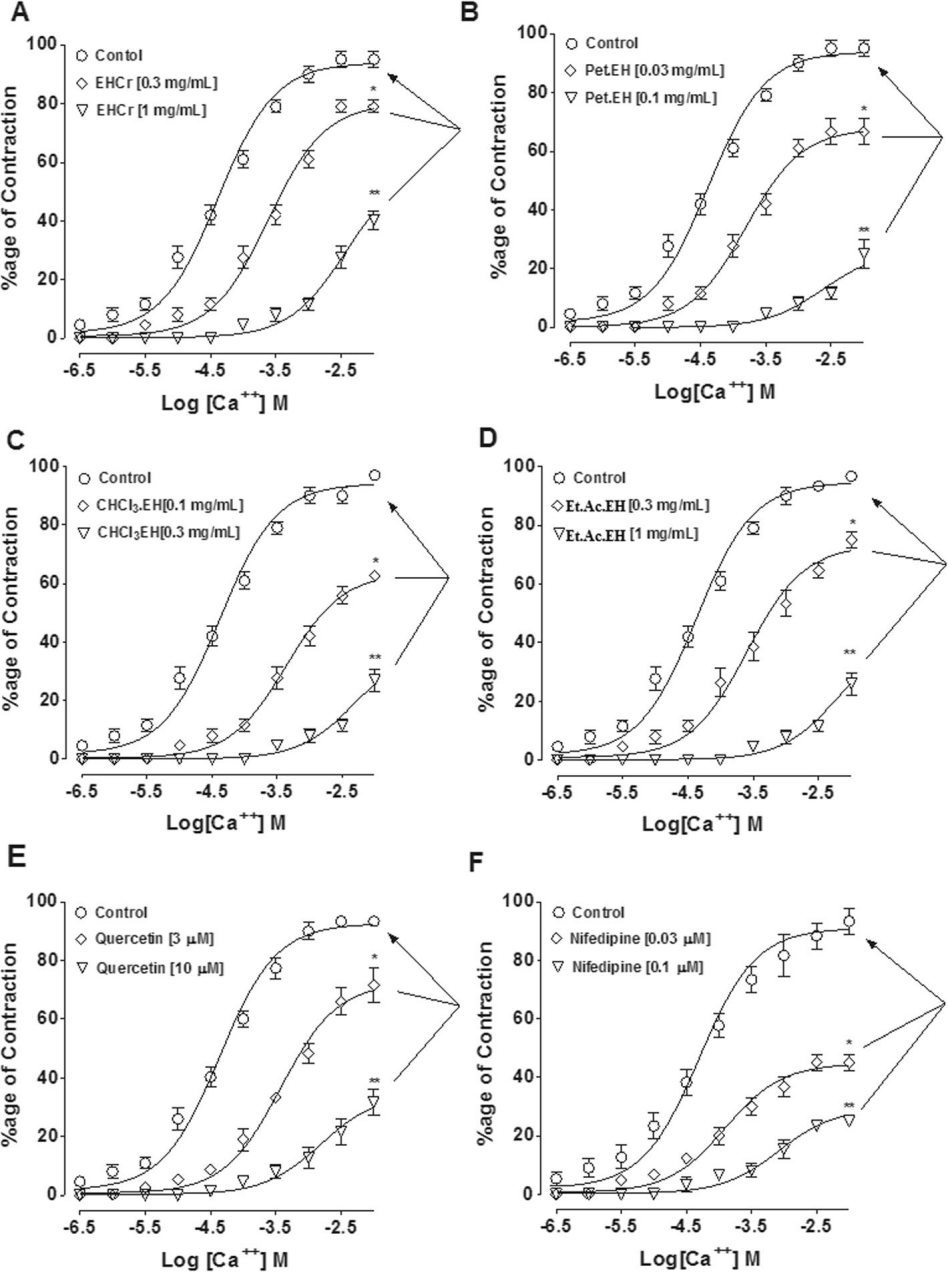
The concentration–response curves of Ca^++^ in the absence and presence of increasing concentrations of the crude extract of *E. hirta* (**a**), its fractions petroleum ether (Pet.EH) (**b**), chloroform (CHCl_3_.EH) (**c**), ethyl acetate (Et.Ac.EH) (**d**), quercetin (**e**) and nifedipine (**f**). *p < 0.05, **p < 0.01 (two-way ANOVA followed by Bonferroni’s post-test correction). The data shown as mean ± S.E.M, *n* = 3-4 individual experiments using tissues of 3 to 4 different animals

**Fig. 6:**
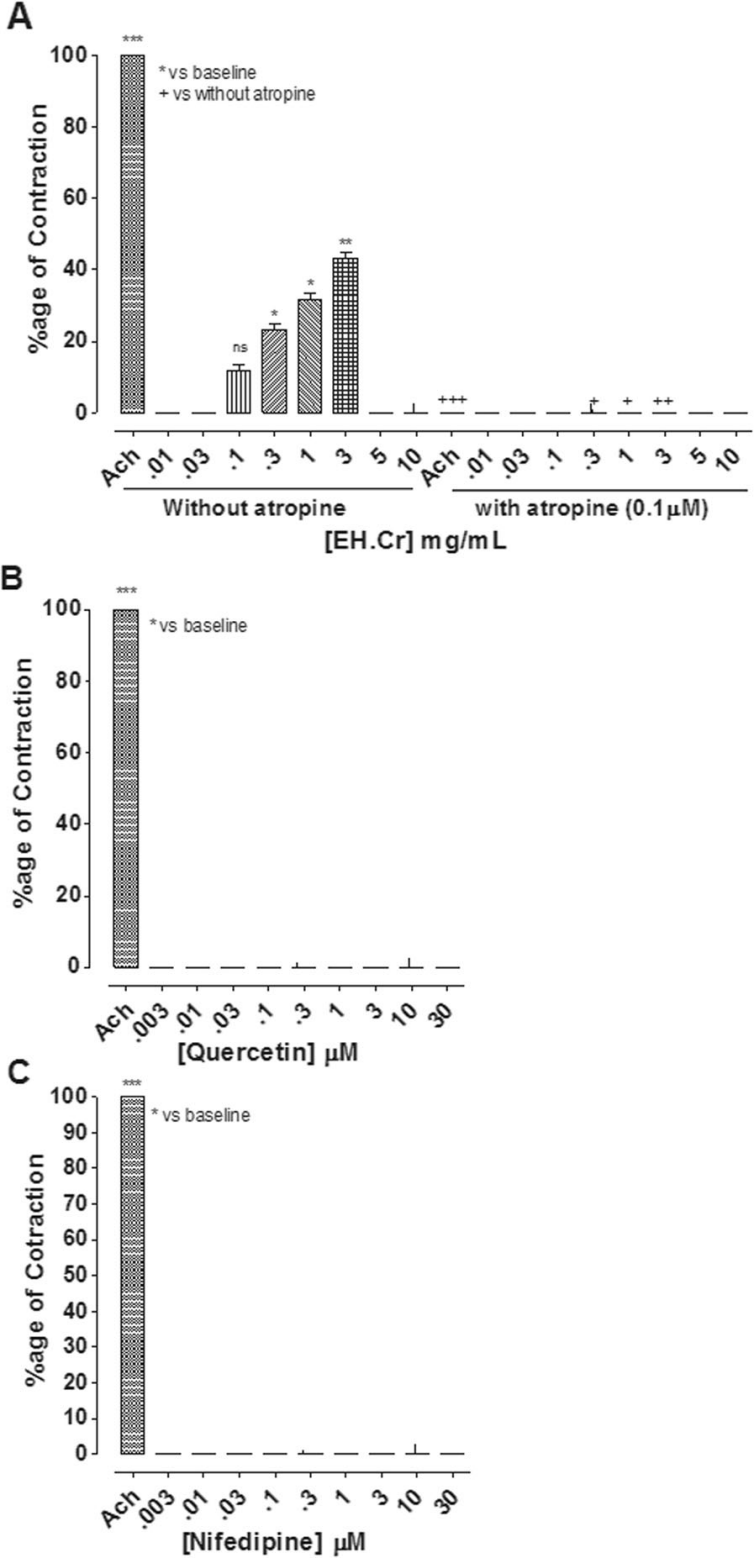
The concentration-dependent excitatory effect of the crude extract of *E. hirta* (**a**) quercetin (**b**) and nifedipine (**c**) without and with atropine (0.1 μM) on isolated rat ileum. {*/^+^*P* < 0.05, **/^++^*P* < 0.01 and ***/^+++^*P* < 0.001] vs. baseline and without atropine responses, ns represents non-significant (One-way ANOVA followed by Dunnet’s test). The data shown as mean ± S.E.M, *n* = 4–6 individual experiments using tissues of 4 to 5 different animals

To test the presence of CCB like activity, the isolated tissues incubated with EH.Cr (0.3 and 1 mg/mL) produced a rightward non-parallel shift in the CRCs of Ca^++^ with significant inhibition of the maximum response of Ca^++^ to 21 ± 2.08% (*n* = 4–6) and 45 ± 2.88% (*n* = 3-5), respectively, similar to the effect of quercetin and nifedipine as seen in Fig. 6. Pre-incubation of tissues with Pet.EH (0.03 and 0.1 mg/mL), CHCl_3_.EH (0.1 and 0.3 mg/mL) and Et.Ac.EH (0.3 and 1 mg/mL) fractions also produced a rightward non-parallel shift in the CRCs of Ca^++^ with marked inhibition of the maximum response of Ca^++^ to 25 ± 5 and 66.6 ± 4.45, 37.6 ± 1.45 and 65 ± 2.90, and 25 ± 2.78 and 62.3 ± 1.66% (*n* = 3-4) respectively, as seen in Fig. 5. The aqueous fraction was devoid of such effect (data not shown).

#### Effect on rat ileum

**Fig. 7:**
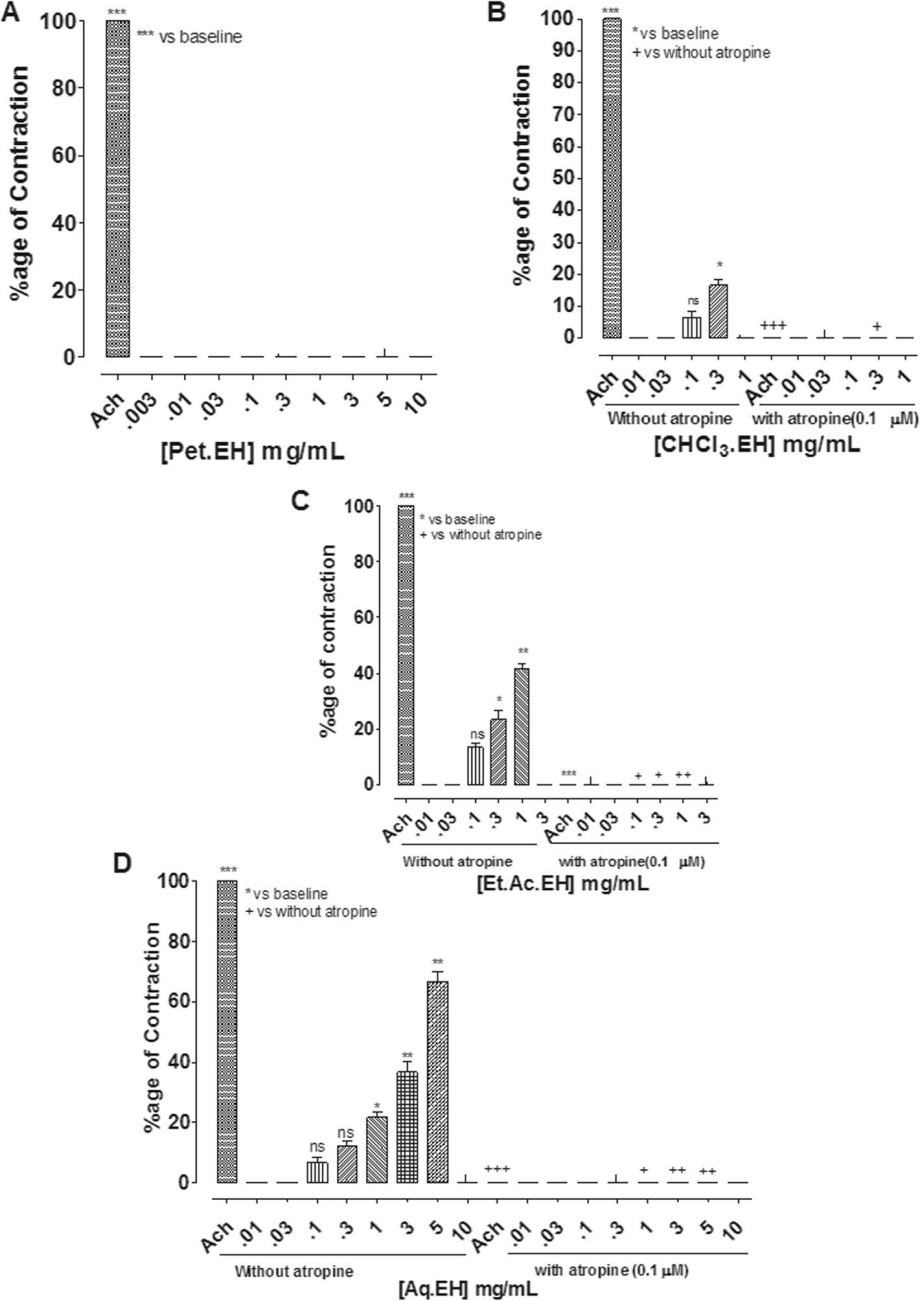
The concentration-dependent excitatory effect of different fractions of *E. hirta*, petroleum ether (Pet.EH) (**a**), chloroform (CHCl_3_.EH) (**b**), ethyl acetate (Et.Ac.EH) (**c**) and aqueous (Aq.EH) (**d**) without and with atropine (0.1 μM) on isolated rat ileum. {*/^+^*P* < 0.05, **/^++^*P* < 0.01 and ***/^+++^*P* < 0.001] vs. baseline and without atropine responses, ns represents non-significant (One-way ANOVA followed by Dunnet’s test). The data shown as mean ± S.E.M, *n* = 4–6 individual experiments using tissues of 4 to 5 different animals

*E. hirta* extract produced a concentration-dependent (0.1–3 mg/mL) excitatory effect with maximum of 43.3 ± 1.66% (*n* = 4–6) contraction relative to Ach (0.3 μM)-mediated excitation. To characterize the excitatory effect of EH.Cr, the rat ileum preparations were pre-treated with atropine (0.1 μM). In the presence of atropine, the excitatory effect of EH.Cr was blocked (Fig. 6a). Quercetin and nifedipine did not exhibit any contractile effect in rat ileum (Fig. 6b, c). Among fractions of *E. hirta,* CHCl_3_.EH, Et.Ac.EH and Aq.EH produced excitatory effects at concentration range of 0.1 and 0.3, 0.1–1 and 0.1–5 mg/mL, respectively, with mean percent maximum of 16.6 ± 1.65, 41.6 ± 1.67 and 66.6 ± 3.33%, respectively. The aqueous fraction was found the most potent. In the presence of atropine, the stimulatory effect of CHCl_3_.EH, Et.Ac.EH and Aq.EH fractions were completely blocked, however, pet-ether fraction did not show any contractile effect in rat ileum (Fig. 7). The gut stimulant effect of the crude extract and its fractions were devoid of any change when restudies in the tissues pretreated with pyrilamine and methysergide (data shown in Additional file 1).

## Discussion

In an attempt to rationalize the medicinal uses of *E. hirta* in diarrhea and constipation, its administration to mice at the dose of 500 and 1000 mg/kg, produced a dose-dependent protection against castor oil-induced diarrhea. *E. hirta* extract was also found safe up to the highest administered dose of 10 g/kg in mice. Castor oil is a triglyceride obtained from the seeds of *Ricinus communis.* After oral administration, ricinoleic acid is released by lipases in the intestinal lumen and induces a strong laxative effect by changing the transport of electrolytes, water and intestinal motility [35]. The determined antidiarrheal effect of *E. hirta* is also in line with the earlier reports indicating its antidiarrheal efficacy in animal models [20, 21].

In naïve and loperamide-fed constipated mice, *E. hirta* at lower doses (50 and 100 mg/kg) caused an increase in total fecal output as well as wet feces, thus providing the first evidence to its folkloric use in constipation. Constipation refers to slow bowel movements (or hypo-mobility), which results in infrequent and/or hard movement of stool, reduced fecal water contents and the attenuated frequency of defecation [36]. Carbachol, a cholinergic drug [37], is known to excite gut musculature through muscarinic receptor activation resulting in increased gut motility, augmented gastrointestinal secretions and enhanced total fecal output including wet feces [38]. Loperamide induces spastic constipation, reduces fecal mass and delays fecal evacuation by inhibiting gut secretions and motility mainly mediating its effect involving opioid receptors [39]. These findings on the part of *E. hirta* in naïve and loperamide-fed constipated mice not only supports its folk use in hypoactive gut disorders but also a further exploration of a preliminary report on *E. hirta* indicating the presence of only in vitro non-specific gut muscle stimulant effects [22]. The prominent laxative effect of *E. hirta* in naïve mice compared to its effect in loperamide-fed constipated mice signifies that the observed laxative effect may involve multiple mechanisms. In the isolated tissues, gut stimulant effect was found primarily mediated through activation of muscarinic receptors, while its effect in loperamide-fed constipated mice, indicated the added involvement of opioid receptors, thus providing as evidence in part to the medicinal use of *E. hirta* in constipation. However, at higher tested dose of 300 mg/kg, EH.Cr produced weaker laxative effect, indicating the stimulant components of the plant co-existed with anti-spasmodic constituents at relatively higher doses. The co-presence of spasmodic and antispasmodic constituents in medicinal plants is usual and has already been reported on the part of other medicinal herbs [1, 3, 40].

Isolated rabbit jejunum produces a regular pendulum-like movement, a steady state of contraction and relaxation. Rabbit jejunum is considered suitable preparation to determine the spasmolytic activity of test material(s) [3, 41]. Spontaneous pendulum-like movements of rabbit jejunum are mediated through an increase in concentration of cytosolic Ca^++^ ultimately resulting in Ca^++^-calmodulin complex mediated depolarization [28]. A test substance causing complete inhibition of spontaneously contracting rabbit jejunum may indicate its ability to interfere with cytosolic Ca^++^ influx. To investigate the possible mechanism of the observed inhibitory effect, the plant material was tested on sustained contraction induced by high [28] and low [42] concentrations of K^+^. High and low K^+^ mediate contractions through the entry of Ca^++^ into muscle cell via voltage dependent Ca^++^ channels (VDCCs) and K^+^ channel opening, respectively. The plant extract and quercetin caused non-specific inhibition of high and low K^+^ − induced contractions, similar to the effect of nifedipine, a known Ca^++^ antagonist [30, 31]. Since high K^+^ is associated with Ca^++^ influx and low K^+^ with K^+^ channel activation, the lack of difference between the two strongly suggests that Ca^++^ antagonism rather than K^+^ out-flux to be the likely mechanism. To further confirm Ca^++^ antagonist like activity, the CRCs of Ca^++^ were constructed in the absence and presence of EH.Cr and quercetin in isolated rabbit jejunum. Incubation of the tissues with EH.Cr and quercetin shifted the CRCs of Ca^++^ to the right by suppressing the maximum response, thus attesting the presence of Ca^++^ antagonist like activity in the plant extract. The effect of EH.Cr was found similar to the activity pattern of nifedipine, a standard CCB, which is known to produce spasmolytic effect on jejunum through blocked of VDCCs. The Ca^++^ channel blockers are known for their antispasmodic and anti-diarrheal effects [43], hence, the presence of Ca^++^ antagonist constituents in *E. hirta* might be contributing to its antidiarrheal effect.

To identify the possible underlying mechanism of gut stimulant effect, *E. hirta* was tested on rat ileum, a semi-quiescent preparation sensitive to spasmodic effects and is considered better compared to rabbit jejunum for the assessment of gut stimulant effects [34, 44]. The crude extract produced stimulant effect through the involvement of muscarinic receptors, which was evident by its complete blockade with atropine, a known cholinergic antagonist [37], thus eliciting the presence of acetylcholine like spasmogenic components in addition to its spasmolytic constituents.

This is first report indicating Ca^++^ antagonist like inhibitory constituents predominantly mediating the antidiarrheal effect of *E. hirta* and also determined its laxative effect mediated primarily through activation of muscarinic receptors. The crude extract of *E. hirta* and its solvent-guided fractions revealed a distribution of spasmolytic and spasmogenic effects when tested on isolated rabbit jejunum and rat ileum. In rabbit jejunum, the crude extract, aqueous and ethyl acetate fractions displayed dual (spasmolytic and spasmogenic) components with varying degrees, which was evident by potentiation of the observed spasmolytic effect when reproduced in the presence of atropine, a cholinergic antagonist [37]. The chloroform and petroleum ether fractions exhibited prominent relaxant effects compared to ethyl acetate fraction while the aqueous fraction was found the weakest in its spasmolytic effect. Thus, indicating that the nonpolar plant components were concentrated in lower polar organic solvent led fractions, showing predominant antispasmodic effects. This is in line with reported literature that alkaloids (ephedrine, morphine and piperine) and flavonoids (quercetin, rutin and kaempferol) are known for their spasmolytic effects and are likely to be soluble in lower polar organic solvents [45, 46]. In rat ileum EH.Cr and its fractions caused gut excitation mediated through the activation of cholinergic pathway with varying potency. The aqueous fraction was found the most potent followed by ethyl acetate and chloroform fractions, while pet-ether fraction was found devoid of any excitatory effect, thus revealing the polar-nature of spasmogenic constituents. It is also reported that saponin, tanins and cardiac glycosides are mostly gut stimulant in nature [47] and are more likely to be soluble in aqueous fraction [48].

The data showed that excitatory effect of *E. hirta* is predominantly mediated through muscarinic receptors activation, which causes an increase in the gut motility directly effecting gut musculature, however the muscarinic agonist(s) are not used to treat constipation due to non-specificity in action which leads to undesirable effects like bradycardia, diarrhea, abdominal cramps, salivation, convulsions and increased urination [49]. *E. hirta* possesses combination of gut stimulant and relaxant components which are installed by nature to antagonize excessive gut stimulant and/or relaxant effects when required or specific expression of constituents (gut stimulant or relaxant) as per disease status (constipation or diarrhea). The co-existence of opposing nature of constituents is commonly observed in ispaghula [1], *Carissa carandas* [3], *Hibiscus rosasinensis* [28] and ginger [40] which indicates the possibility of synergistic and/or adverse effect overcoming combinations in same remedy.

Quercetin, a flavonoid constituent, was found in relatively high proportion in *E. hirta* compared to other standards which was evident by HPLC chromatogram. Quercetin also showed antispasmodic and antidiarrheal effects. In addition, *E. hirta* is reported to contain kaempferol, β-sitosterol, and rutin with known spasmolytic activities [3, 44, 50]. This plant is also known to contain caffeic acid and epicatechin 3-gallate acid, which are reported as anti-microbial [11]. The presence of these phytochemicals with anti-microbial potential in this plant supports its medicinal use in diarrhea. This preclinical study provides a way forward for further clinical and isolation studies on *E. hirta* for its true translational impact and isolation of newer phyto-based chemical moieties to combat diarrhea and/or constipation.

## Conclusion

This study showed that *E. hirta* possesses antidiarrheal and laxative activities. The antidiarrheal effect was mediated through gut inhibitory (Ca^++^ antagonist), while the laxative effect was derived through gut excitatory components causing activation of muscarinic receptors. Fractionation revealed that the petroleum ether fraction acts exclusively through Ca^++^ antagonist like mechanism, the aqueous fraction predominantly involves cholinergic receptor agonist like pathway, while the others fractions showed dual (spasmodic and antispasmodic) effects. Thus, these findings provide a rationale to the folk use of *E. hirta* in diarrhea and constipation.

## Supplementary information

**Additional file 1.** Raw data representing the antispasmodic, Ca^++^ antagonist and spasmodic effects of the crude extract of *E. hirta* and its fractions on naïve and spasmogen challenged [low (20 mM) and high (80 mM) K^+^] isolated rabbit jejunum and naïve rat ileum.

## Data Availability

The datasets used and/or analyzed during the current study are made available as supplementary files.

## Abbreviations

Ach: Acetylcholine
Aq.EH: Water fraction of *Euphorbia hirta*
Ca^++^: Calcium
CCBs: Calcium channel blockers
CCh: Carbachol
CHCl_3_.EH: Chloroform fraction of *Euphorbia hirta*
CRCs: Concentration response curves
*E. hirta*: *Euphorbia hirta*
EH.Cr: *Euphorbia hirta* crude extract
Et.Ac.EH: Ethyl acetate fraction of *Euphorbia hirta* and
Pet.EH: Petroleum-ether fraction of *Euphorbia hirta*
VDCCs: Voltage dependent Ca^++^ channels
