# Supplementary material for: The use of Euphorbia hirta L. (Euphorbiaceae) in diarrhea and constipation involves calcium antagonism and cholinergic mechanisms
Source: BMC Complement Med Ther. 2020 Jan 16;20:14. doi: 10.1186/s12906-019-2793-0 (PMC7076812; doi:10.1186/s12906-019-2793-0)
Supplement: Supplementary file 1 — Additional file 1. Raw data representing the antispasmodic, Ca++ antagonist and spasmodic effects of the crude extract of E. hirta and its fractions on naïve and spasmogen challenged [low (20 mM) and high (80 mM) K+] isolated rabbit jejunum and naïve rat ileum. [file 12906_2019_2793_MOESM1_ESM.docx]

| EH.Cr | Without atropine - percent response | | | | | | | | With atropine -percent response | | | | | | | |
| --- | --- | --- | --- | --- | --- | --- | --- | --- | --- | --- | --- | --- | --- | --- | --- | --- |
| Conc. | 1 | 2 | | 3 | 4 | | 5 | 6 | 1 | 2 | 3 | | 4 | | 5 | |
| 0.01  0.03  0.10  0.30  1.00  3.00  5.00  10.00 | 100.  100.  100.  100.  90.  80.  40.  0. | | 100.  100.  100.  100.  90.  80.  60.  0. | 100.  100.  100.  100.  100.  90.  40.  0. | | 100.  100.  100.  100.  90.  80.  50.  0. | 100.  100.  100.  100.  95.  85.  40.  0. | 100  100  100  100  98  84  45  0 | 100  100  100  90  80  40  0 | 100  100  100  90  80  60  0 | | 100  100  100  100  90  40  0 | | 100  100  100  90  80  40  0 | | 100  100  100  95  80  50  0 |

**Raw DATA of *E. hirta* on rabbit jejunum and rat ileum**

The inhibitory effect of EH.Cr against rabbit jejunum in absence and presence of atropine. n = 4-6 individual experiments using tissues of 4 to 5 different animals.

The inhibitory effect of EH.Cr against high K^+^ and low K^+^ induced contraction in jejunum. n = 4-6 individual experiments using tissues of 4 to 5 different animals.

| EH.Cr | High K^+^ - percent response | | | | | | | Low K^+^ - percent response | | | | | |
| --- | --- | --- | --- | --- | --- | --- | --- | --- | --- | --- | --- | --- | --- |
| Conc. | 1 | 2 | | 3 | 4 | | 5 | 1 | 2 | 3 | 4 | 5 | 6 |
| 0.01  0.03  0.10  0.30  1.00  3.00  5.00 | 100.  100.  100.  90.  60.  0.  0. | | 100.  100.  100.  80.  80.  10.  0. | 100  100  95  95  80  7  0 | | 100.  100.  100.  85.  80.  10.  0. | 100  100  96  95  75  5  0 | 100  100.  90.  85.  80.  0.  0. | 100  100  100  90  60  10  0 | 100  100  100  80  65  0  0 | 100  100  100  86  55  8  0 | 100  100  100  80  65  0  0 | 100  100  95  87  60  7  0 |

The inhibitory effect of Pet.EH against rabbit jejunum in absence and presence of atropine. n = 4-6 individual experiments using tissues of 4 to 5 different animals.

| Pet.EH | Without atropine - percent response | With atropine - percent response |
| --- | --- | --- |
| Conc. | 1 2 3 4 5 6 | 1 2 3 4 5 |
| 0.003  0.010  0.030  0.100 | 100 100 90 100 95 100  90 80 70 90 80 95  70 60 60 70 70 72  0 0 0 0 0 0 | 95 100 100 100 100  90 90 90 80 90  65 65 70 60 70  0 0 0 0 0 |

The inhibitory effect of Pet.EH against high K^+^ and low K^+^ induced contraction in jejunum. n = 4-6 individual experiments using tissues of 4 to 5 different animals.

| Pet EH | High K^+^ | Low K^+^ |
| --- | --- | --- |
| n | 1 2 3 4 5 6 | 1 2 3 4 5 |
| 0.001  0.003  0.010  0.030  0.100 | 100 100 100 100 100 100  100 100 90 100 95 99  80 80 70 85 75 82  70 60 60 75 65 60  0 0 0 0 0 0 | 100 100 100 100 100  90 100 100 95 100  80 90 90 85 95  60 70 70 65 75  0 0 0 0 0 |

The inhibitory effect of CH_3_Cl.EH against rabbit jejunum in absence and presence of atropine. n = 4-6 individual experiments using tissues of 4 to 5 different animals.

| CHCl_3_. EH | Without atropine - percent response | With atropine - percent response |
| --- | --- | --- |
| Conc. | 1 2 3 4 5 6 | 1 2 3 4 5 |
| 0.01  0.03  0.10  0.30  1.00  3.00 | 100 100 100 100 100 100  100 90 95 90 100 90  85 75 75 75 85 80  60 60 50 60 60 55  0 10 22 12 25 20  0 0 0 0 0 0 | 100 100 100 100 100  95 100 95 100 90  70 85 75 80 80  55 65 45 60 60  0 10 0 5 5  0 0 0 0 0 |

The inhibitory effect of CH_3_Cl.EH against high K^+^ and low K^+^ induced contraction in jejunum. n = 4-6 individual experiments using tissues of 4 to 5 different animals.

| CHCl_3_. EH | High K^+^ - percent response | Low K^+^ - percent response |
| --- | --- | --- |
| Conc | 1 2 3 4 5 | 1 2 3 4 5 6 |
| 0.01  0.03  0.10  0.30  1.00 | 100 100 100 100 100  95 100 100 100 89  90 80 70 80 78  70 50 40 45 47  0 0 0 0 0 | 100 100 100 100 100 100  100 90 90 95 90 92  80 70 80 70 75 80  40 50 60 45 40 41  0 0 0 0 0 0 |

The inhibitory effect of Et.Ac.EH against rabbit jejunum in absence and presence of atropine. n = 4-6 individual experiments using tissues of 4 to 5 different animals.

| ET.Ac.EH | With atropine - percent response | Without atropine - percent response |
| --- | --- | --- |
| Conc | 1 2 3 4 5 6 | 1 2 3 4 |
| 0.01  0.03  0.10  0.30  1.00  3.00  5.00 | 100 100 100 100 100 100  100 100 100 100 100 100  100 100 100 100 100 100  90 90 80 95 90 90  70 70 65 70 60 65  20 50 30 30 25 29  0 0 0 0 0 0 | 100 100 100 100  100 100 100 100  100 100 90 95  80 90 70 85  60 80 60 70  0 0 0 0 |

The inhibitory effect of Et.Ac.EH against high K^+^ and low K^+^ induced contraction in jejunum. n = 4-6 individual experiments using tissues of 4 to 5 different animals.

| Et.Ac.EH | High K^+^ - percent response | Low K^+^ - percent response |
| --- | --- | --- |
| Conc. | 1 2 3 4 5 6 | 1 2 3 4 |
| 0.01  0.03  0.10  0.30  1.00  3.00 | 100 100 100 100 100 100  100 100 100 100 100 99  90 100 100 100 95 90  80 90 80 80 85 75  60 50 60 50 40 35  0 0 0 0 0 0 | 100 100 100 100  100 100 90 100  90 100 85 100  70 90 70 80  50 60 50 70  0 0 0 0 |

The inhibitory effect of Aq.EH against rabbit jejunum in absence and presence of atropine. n = 4-6 individual experiments using tissues of 4 to 5 different animals.

| Aq.EH | Without atropine - percent response | With atropine - percent response |
| --- | --- | --- |
| Conc | 1 2 3 4 5 6 | 1 2 3 4 5 |
| 0.01  0.03  0.10  0.30  1.00  3.00  5.00  10.00 | 100 100 100 100 100 100  100 100 100 100 100 100  100 100 100 100 100 100  100 100 100 100 100 100  90 100 100 100 90 100  70 100 90 100 70 90  60 70 60 70 60 60  30 40 40 40 30 40 | 100 100 100 100 100  100 100 100 100 100  100 100 100 100 100  100 90 100 100 90  100 75 90 90 75  70 60 50 65 55  0 0 0 0 0 |

The inhibitory effect of Aq.EH against high K^+^ and low K^+^ induced contraction in jejunum. n = 4-6 individual experiments using tissues of 4 to 5 different animals.

| Aq.EH | High K^+^ - percent response | Low K^+^ - percent response |
| --- | --- | --- |
| Conc | 1 2 3 4 5 6 | 1 2 3 4 |
| 0.30  1.00  3.00  5.00  10.00 | 100 100 100 100 100 100  100 100 90 95 90 99  90 90 80 85 80 89  70 80 75 80 70 78  60 70 70 80 60 70 | 100 100 100 100  100 90 90 95  90 85 80 75  70 70 60 55  60 70 60 55 |

**Rat Ileum Data**

Spasmogenic effects of EH.Cr on rat ileum in presence and absence of atropine. n = 4-6 individual experiments using tissues of 4 to 5 different animals.

| EH.Cr | Without atropine - percent response | | | | | | With atropine - percent response |
| --- | --- | --- | --- | --- | --- | --- | --- |
| Conc | 1 2 3 4 5 6 | | | | | | 1 2 3 4 |
| Ach  0.01  0.03  0.10  0.30  1.00  3.00  5.00  10.00 | 100  0  0  10  20  30  40  0  0 | 100  0  0  15  25  30  45  0  0 | 100  0  0  10  25  35  45  0  0 | 100  0  0  15  25  30  45  0  0 | 100  0  0  8  23  33  42  0  0 | 100  0  0  10  20  25  40  0  0 | 0 0 0 0  0 0 0 0  0 0 0 0  0 0 0 0  0 0 0 0  0 0 0 0  0 0 0 0  0 0 0 0  0 0 0 0 |

Spasmogenic effects of EH.Cr on rat ileum in presence and absence of atropine. n = 4-6 individual experiments using tissues of 4 to 5 different animals.

| CH_3_Cl.EH | Without atropine - percent response | | | | | | With atropine - percent response |
| --- | --- | --- | --- | --- | --- | --- | --- |
| Conc | 1 2 3 4 5 6 | | | | | | 1 2 3 4 |
| Ach  0.01  0.03  0.10  0.30  1.00  3.00  5.00  10.00 | 100  0  0  5  20  0  0  0  0 | 100  0  0  10  15  0  0  0  0 | 100  0  0  5  15  0  0  0  0 | 100  0  0  7  15  0  0  0  0 | 100  0  0  5  18  0  0  0  0 | 100  0  0  12  20  0  0  0  0 | 0 0 0 0  0 0 0 0  0 0 0 0  0 0 0 0  0 0 0 0  0 0 0 0  0 0 0 0  0 0 0 0  0 0 0 0 |

Spasmogenic effects of Et.Ac.EH on rat ileum in presence and absence of atropine. n = 4-5 individual experiments using tissues of 4 to 5 different animals.

| Et.Ac.EH | Without atropine - percent response | | | | | | With atropine - percent response |
| --- | --- | --- | --- | --- | --- | --- | --- |
| Conc. | 1 2 3 4 5 6 | | | | | | 1 2 3 4 |
| Ach  0.01  0.03  0.10  0.30  1.00  3.00  5.00  10.00 | 100  0  0  10  20  40  0  0  0 | 100  0  0  15  30  45  0  0  0 | 100  0  0  15  20  45  0  0  0 | 100  0  0  15  30  45  0  0  0 | 100  0  0  12  17  42  0  0  0 | 100  0  0  8  20  39  0  0  0 | 0 0 0 0  0 0 0 0  0 0 0 0  0 0 0 0  0 0 0 0  0 0 0 0  0 0 0 0  0 0 0 0  0 0 0 0 |

Spasmogenic effects of Aq.Cr on rat ileum in presence and absence of atropine. n = 4-5 individual experiments using tissues of 4 to 5 different animals.

| Aq.EH | Without atropine - percent response | | | | | | With atropine - percent response |
| --- | --- | --- | --- | --- | --- | --- | --- |
| Conc. | 1 2 3 4 5 | | | | | 6 | 1 2 3 4 |
| Ach  0.01  0.03  0.10  0.30  1.00  3.00  5.00  10.00 | 100  0  0  5  10  20  60  0  0 | 100  0  0  5  15  20  70  0  0 | 100  0  0  10  12  25  70  0  0 | 100  0  0  13  13  25  70  0  0 | 100  0  0  0  12  18  63  0  0 | 100  0  0  0  10  17  60  0  0 | 0 0 0 0  0 0 0 0  0 0 0 0  0 0 0 0  0 0 0 0  0 0 0 0  0 0 0 0  0 0 0 0  0 0 0 0 |

**Calcium curve**

Concentration response curves of EH.Cr on rabbit jejunum.

| Log[Ca^++^] | Control - percent response | EHCr[0.3mg/mL] - percent response | EHCr[1mg/mL] - percent response |
| --- | --- | --- | --- |
| Conc | 1 2 3 4 | 1 2 3 4 | 1 2 3 4 5 |
| -6.5  -6.0  -5.5  -5.0  -4.5  -4.0  -3.5  -3.0  -2.5  -2.0 | 0 4. 8. 0  6. 8. 12. 9  10. 12. 15. 25  15. 30. 33. 30  30. 45. 46. 40  50. 63. 65. 68  70. 80. 82. 87  80. 95. 90. 90  90. 95. 95. 95  95. 95. 95. 100 | 0. 0. 0. 0  0. 0. 0. 0  2. 4. 8. 0  4. 8. 12. 5  8. 12. 15. 10  20. 30. 33. 25  35. 45. 46. 40  55. 63. 65. 59  75. 80. 82. 88  75. 80. 82. 90 | 0. 0. 0. 0 0  0. 0. 0. 0 0  0. 0. 0. 0 0  0. 0. 0. 0 0  0. 0. 0. 0 0  2. 4. 8. 6 10  4. 8. 12. 10 15  8. 12. 15. 20 25  20. 30. 33. 27 30  35. 40. 46. 40 45 |

Concentration response curves of Pet.EH on rabbit jejunum.

| Log[Ca^++^] | Control - percent response | | |  | Pet.EH [0.03mg/mL] - percent response | | | | Pet.EH [0.1mg/mL] - percent response |
| --- | --- | --- | --- | --- | --- | --- | --- | --- | --- |
| Conc. | 1 | 2 | 3 | 4 | 1 2 3 4 | | | | 1 2 3 |
| -6.5  -6.0  -5.5  -5.0  -4.5  -4.0  -3.5  -3.0  -2.5  -2.0 | 2  4  8  20  35  55  75  85  90  90 | 4  8  12  30  45  63  80  95  95  95 | 8  12  15  33  46  65  82  90  95  95 | 10  15  18  30  38  59  70  78  90  95 | 0  0  0  4  8  20  35  55  60  60 | 0  0  0  8  12  30  45  63  75  75 | 0  0  0  12  15  33  46  65  65  65 | 0  0  0  10  18  30  40  60  60  61 | 0 0 0  0 0 0  0 0 0  0 0 0  0 0 0  0 0 0  2 4 8  4 8 12  8 12 15  15 30 30 |

Concentration response curves of CHCl_3_.EH on rabbit jejunum.

| Log[Ca^++^] | Control - percent response | | | | EH.CHCl_3_[0.1mg/mL] - percent response | | | EH.CHCl_3_ [0.3mg/mL]  - percent response | | | |
| --- | --- | --- | --- | --- | --- | --- | --- | --- | --- | --- | --- |
| Conc. | 1 | 2 | 3 | 4 | 1 | 2 | 3 | 1 | 2 | 3 |  |
| -6.5 | 2 | 4 | 8 | 0 | 0 | 0 | 0 | 0 | 0 | 0 |  |
| -6.0 | 4 | 6 | 12 | 10 | 0 | 0 | 0 | 0 | 0 | 0 |  |
| -5.5 | 8 | 15 | 15 | 12 | 0 | 0 | 0 | 0 | 0 | 0 |  |
| -5.0 | 20 | 25 | 33 | 28 | 2 | 4 | 8 | 0 | 0 | 0 |  |
| -4.5 | 35 | 40 | 46 | 30 | 4 | 8 | 12 | 0 | 0 | 0 |  |
| -4.0 | 55 | 60 | 65 | 62 | 8 | 12 | 15 | 0 | 0 | 0 |  |
| -3.5 | 75 | 85 | 82 | 80 | 20 | 30 | 33 | 2 | 4 | 8 |  |
| -3.0 | 85 | 95 | 90 | 96 | 35 | 45 | 46 | 4 | 8 | 12 |  |
| -2.5 | 90 | 95 | 95 | 98 | 50 | 60 | 58 | 8 | 12 | 15 |  |
| -2.0 | 90 | 95 | 95 | 98 | 60 | 65 | 63 | 20 | 28 | 33 |  |
|  |  |  |  |  |  |  |  |  |  |  |  |

Concentration response curves of Et.Ac.EH on rabbit jejunum.

| Log[Ca^++^] | Control - percent response | | | | EtAt.EH [0.3mg/mL] - percent response | | | Et.At.EH [1mg/mL] - percent response | | |
| --- | --- | --- | --- | --- | --- | --- | --- | --- | --- | --- |
| Conc | 1 | 2 |  | 3 | 1 | 2 | 3 | 1 | 2 | 3 |
| -6.5  -6.0  -5.5  -5.0  -4.5  -4.0  -3.5  -3.0  -2.5  -2.0 | 2  4  8  20  35  55  75  85  90  90 | 4  8  12  30  45  63  80  95  95  95 | 6  10  12  35  40  60  85  90  90  90 |  | 0  0  2  4  8  18  30  45  60  70 | 0  0  4  8  12  26  40  55  66  80 | 0  0  8  12  15  35  46  60  68  75 | 0  0  0  0  0  0  2  4  8  20 | 0  0  0  0  0  0  4  8  12  25 | 0  0  0  0  0  0  8  12  15  33 |

Concentration response curves of Quercetin on rabbit jejunum.

| Log[Ca^++^] | Control - percent response | | | Quercetin[3 µM] - percent response | | | | Quercetin [10 µM] - percent response | | |
| --- | --- | --- | --- | --- | --- | --- | --- | --- | --- | --- |
| Conc. | 1 | 2 | 3 | 1 | 2 | 3 | 4 | 1 | 2 | 3 |
| -6.5  -6.0  -5.5  -5.0  -4.5  -4.0  -3.5  -3.0  -2.5  -2.0 | 2  4  8  20  35  55  75  85  90  90 | 4  6  10  25  40  60  75  90  90  95 | 8  12  15  33  46  65  82  90  95  95 | 0  0  2  4  8  20  35  55  75  75 | 0  0  0  4  8  12  30  45  63  80 | 0.  0  6  8  10  25  35  45  60  60 | 0  0  10  12  15  29  33  40  60  75 | 0  0  0  0  0  2  4  8  15  25 | 0  0  0  0  4  8  12  20  30  40 | 0  0  0  0  0  4  8  10  20  30 |

Concentration response curves of Nifedipine on rabbit jejunum.

| Log[Ca^++^] | Control | | | Nifedipine[0.1µM] | Nifedipine[0.3µM] |
| --- | --- | --- | --- | --- | --- |
| n | 1 | 2 3 4 | | 1 2 3 | 1 2 3 |
| -6.5  -6.0  -5.5  -5.0  -4.5  -4.0  -3.5  -3.0  -2.5  -2.0 | 2  6  8  15  30  50  70  80  85  90 | 4  8  12  30  45  63  80  95  95  95 | 8 10  12 15  15 19  33 25  46 35  65 40  82 85  90 90  95 95  95 99 | 0 0 0  0 0 0  2 4 8  4 6 10  10 12 15  15 20 25  30 25 35  40 30 40  45 40 50  45 40 50 | 0 0 0  0 0 0  0 0 0  0 0 0  0 2 8  4 6 10  4 8 12  15 10 21  20 25 25  25 25 25 |
